# Sports Courage in Malaysian Silat Athletes: Confirmatory Factor Analysis of the Malay Language Version

**DOI:** 10.3390/ijerph17051736

**Published:** 2020-03-06

**Authors:** Aizuddin Bin Hidrus, Yee Cheng Kueh, Wan Nor Arifin, Erkut Konter, Garry Kuan

**Affiliations:** 1 Unit of Biostatistics and Research Methodology, School of Medical Sciences, Universiti Sains Malaysia, Kubang Kerian 16150, Kelantan, Malaysia; aizuddinh88@gmail.com (A.B.H.); wnarifin@usm.my (W.N.A.); 2 Community and Family Medicine Department, Faculty of Medicine and Health Science, Universiti Malaysia, Kota Kinabalu 88400, Sabah, Malaysia; 3 Buca Educational Faculty, Dokuz Eylül University, 35220 Izmir, Turkey; erkut.konter@gmail.com; 4 Exercise and Sports Science Programme, School of Health Sciences, Universiti Sains Malaysia, Kubang Kerian 16150, Kelantan, Malaysia; 5 Department of Life Sciences, Brunel University, London UB8 3PH, UK

**Keywords:** sports courage, Silat athletes, confirmatory factor analysis, Malay language

## Abstract

Sports courage is one of the most important attributes to help competitive athletes overcome anxiety, nervousness, and other psychological obstacles, but this field of study is still being overlooked by most athletes and coaches. The purpose of this study is to validate the Malay language version of the Sports Courage Scale (SCS-M) for Silat athletes using confirmatory factor analysis (CFA). Data were collected during 9th UPSI International Pencak Silat Championship in Malaysia. A total of 258 competitors (male = 66.7%, female = 33.3%), with a mean age of 18 years (SD = 2.6), volunteered to participate in this study. The original SCS with 50 items underwent forward and backward translations into the Malay language and was pre-tested with ten martial arts athletes. Then, Silat athletes were asked to complete the translated SCS-M questionnaire. There were five factors in the SCS-M (i.e., mastery, determination, assertiveness, venturesome, and self-sacrificial behaviour). The first hypothesised model with 50 items did not result in a good fit to the data (RMSEA = 0.06, CFI = 0.93, NFI = 0.87, NNFI = 0.93, RMR = 0.14, SRMR = 0.09). A total of 17 problematic items were identified and were removed iteratively. The final measurement model with 33 items fit the data well (RMSEA = 0.06, CFI = 0.94, NFI = 0.89, NNFI = 0.94, RMR = 0.05, SRMR = 0.07). The reliability of each subscale based on Cronbach’s alpha ranged from 0.64 to 0.76. The convergent and discriminant validities were achieved for the final measurement model. The revised version of SCS-M with 33 items was considered valid and reliable for measuring the sports courage in Silat athletes in Malaysia.

## 1. Introduction

Sports courage is one of the most critical attributes in sport psychology. Recently, numerous researchers have proposed different definitions of courage [1]. Kilmann, O’Hara, and Strauss [2], who conducted a review on the factors affecting courage, implied that courageous actions consisted of five essential properties: (1) Free choice in deciding whether to act versus being coerced, (2) significant risk of being harmed, (3) assessment that the risk is reasonable and the act is considered justifiable (not foolhardy), (4) the pursuit of worth, and (5) proceeding with mindful behaviour despite being fearful.

There was a time when controversial issues arose with minimal discussions about courage being viewed as a remedy for treating fear in sport [3]. Inspired by ancient Greek philosophers, such as Plato and Aristotle, Corlett [3] stated that ‘courage was a part of virtuous living’. It was believed that courage, which was originated and used as an emblem of virtue in ancient Greece, had vanished in the modern sport [3]. Therefore, he suggested two ways for sport pedagogues to consider how courage could address fear in athletes: (1) Viewing sports ‘as a social practice worthy of true courage’, and (2) Correcting the handling of athletes’ fear with conventional psychological methods. The in-depth review depicts how courage could significantly advance athletes to perform at higher levels in competitions.

Despite numerous studies being conducted on the meaning of courage in different fields, such as business, leadership, and musical performance, we found limited studies on sports courage among athletes in the literature [4]. Sports courage was recently defined by Konter et al. [5] as a “natural and developed, interactional, and perceptual concept between person and situation, and the task at hand that enables the person to move with competence, mastery, determination, assertiveness, venturesome and sacrificial (altruistic) behaviour on a voluntary basis and in dangerous circumstances”. According to Konter and Ng [4], ‘athletes have been known to display many forms of courage by virtue of their basic human behaviour, intellectual, cognitive, physical, emotional and social fortitude, and resolve while taking care of their opponents (sportsmanship)’. Hence, after conducting several reviews on the importance of sports courage, Konter and Ng [4] developed the Sports Courage Scale (SCS). It was created by recruiting 843 athletes from different sports clubs and schools. All the collected data were then analysed by three different stages, which includes exploratory factor analysis (EFA), confirmatory factor analysis (CFA), and then test-retest reliability, which was reported as interclass correlation coefficient (ICC). The questionnaire consisted of five factors: (1) Mastery (self-confidence), (2) Determination, (3) Assertiveness, (4) Venturesome (coping with fear, risk-taking), and (5) Self-sacrificial behaviour (altruism).

Pencak Silat is considered to be one of the most popular martial arts sports in Malaysia. Most Malay children are exposed to and know about Silat in primary school, both as part of the school curriculum and during after school activities. Students are also able to participate in private Silat lessons at the Silat clubs, associations, or academies. Silat is also a growing popular sport in the Western region [6,7] and other parts of Asia. Moreover, Silat has already earned its recognition in international competitions, such as the World Championship [8,9]. According to Aziz and his colleagues [8], ‘historically, Silat was practised by the native people during their struggles against the colonial rulers’. As time passed, Silat became one aspect of the Malaysian culture commonly used for ceremonial, performance, sport, and recreational purposes, which has been transformed into a structured combat sport [8].

In Malaysia, Silat is an important sport that has achieved many medals in mega-sport events, such as the SEA Games, Commonwealth Games, and Pencak Silat World Championship. Many initiatives have been carried out to help national Silat athletes improve their performance in competitions. More coaches are beginning to realise that improving Silat athletes’ courage levels helps them perform better during competitions.

Presently, the SCS, validated by Konter and Ng [4], is only available in the English and Turkish languages. There is a lack of information about sports courage among Malaysian athletes due to a void in the Malaysian language to measure sports courage. We propose that enhancing sports courage among athletes could be an effective way to increase sports performance, and a validated Malay version would be beneficial for coaches and sport psychologists to measure sports courage in athletes. Thus, the present study is to validate the translated Malay version of the SCS among Malaysian Silat athletes competing in national level open competitions. As sports psychology is still relatively new in Malaysia, the validity and reliability of the Malay version of the SCS are necessary for the applied field. Thus, knowing the level of the athletes’ courage could help them to improve their confidence levels and to boost their spirit in sports. Hence, the main purpose of the present study is to validate the Malay language version of the SCS (SCS-M) questionnaires and to determine the correlations between subscales of SCS-M among the Malaysian Silat athletes who competed in national tournaments.

## 2. Materials and Methods

### 2.1. Participants

A total of 258 Malaysian Silat athletes (Male 66.7%, n = 172; Female 33.3%, n = 86) who participated at the 9th UPSI International Pencak Silat Championship, volunteered and agreed to participate in this study. Their mean age was 18 years old (SD = 2.6), and most of them were Malay (95.3%). They were divided into distinct categories; teenagers, adults, and International open, by which all of them representing their states or clubs. All of the athletes were Malaysian who were able to write, read and speak the Malay language.

### 2.2. Translation of the Questionnaire

The original English version of the SCS was translated into the Malay language using the forward and backward standardised procedures outlined by Brislin [10]. The steps include: (1) The first author forward translated the English version into Malay language, based on the principle of retaining meaning, rather than literal word-for-word translations, (2) two local Malays, who were bilingual in Malay and English, back-translated the Malay version to English, (3) five panels consisted of experts from the areas of sport psychology, health psychology, physical activity, psychometrics, and a language teacher with over ten years of experience in their areas of expertise, reviewed and examined the forward and backward translated versions. All panels were competent bilingual speakers in both Malay and English. They reviewed the English translation from Malay, and Malay translation from English, comparing each item to the corresponding item on the original English version. They noted any deviations in meaning and finalised the Malay version of SCS. Then, expert panels were asked to assess whether the contents were culturally appropriate to the Malaysian population. The final version of SCS-50 Malay version was pre-tested among ten martial arts athletes for clarity and comprehension. The athletes were asked to answer the questions and comment on the wording and the presentation of the questionnaire. We found the result of the pre-test to be good, and no modification was necessary. Thus, the final version of SCS-50 Malay version was used for the validation study.

### 2.3. Data Collection

We began the data collection after receiving the ethical approval from the Human Research Ethics Committee of the Universiti Sains Malaysia (JEPeM Code: USM/JEPeM/16070228). Then, permission was obtained to conduct the data collection from the director of the 9th UPSI International Pencak Silat Championship. The cross-sectional study design was employed on the self-administered SCS-50 questionnaire. The questionnaire was distributed to the athletes a day before the tournament, and the athletes were briefed regarding the study prior to the distribution. The information to Participants’ sheet and SCS-50 questionnaire were distributed to the athletes. Implied consent was obtained when the participants volunteered to complete and return the SCS-50 questionnaire to the researchers. A total of 258 athletes completed the questionnaires and returned them to the researchers.

### 2.4. Measure

#### 2.4.1. Demographic Information

Several demographic questions were administered. These questions assessed the personal attributes of the participants (including age, gender, and ethnicity).

#### 2.4.2. Sport Courage Scale

The SCS is considered as the latest developed instrument for measuring the courage level of the athletes. The original scale consisted of 50 items that are measuring five different subscales, namely; mastery, determination, assertiveness, venturesome, and self-sacrificial behaviour. Each item was scored using five-point Likert-scale from 1 (strongly disagree) to 2 (strongly agree). Based on the literature, the published validation study on the original English version of SCS, the original 50 items were reduced to 31 items after gone through CFA. In the final CFA model, it consists of 31 items with factor loadings vary from 0.43 to 0.78 and also showed good fit: χ^2^ (429) = 584.32, *p* < 0.01, CFI = 0.93, TLI = 0.93, RMSEA = 0.03, SRMR = 0.06 [4]. Cronbach alpha for each subscale was ranged from 0.61 to 0.82. As for the present study, the translated version of SCS-M with 50 items was used. We used SCS-M with 50 items rather than with 31 items because the scale is relatively new as sport-related measurement, especially for Malaysia’s athletes. We expected problematic items would be identified during the analysis. Therefore, more items on the scale are preferred to allow researchers to identify the best items that fit the measurement model during the analysis. The SCS-M is available upon request from the authors.

### 2.5. Statistical Analysis

SCS-M was analysed by CFA using LISREL version 9.1 and SPSS 24 software. It was suggested that for researchers to present more than one fit index in their reports or studies [11]. Several fit indices were used as a guideline to identify best-fit measurement model. According to Hair, Anderson, Babin, and Black [12], the cut-off values for recommended fit indices are: the comparative fit index (CFI) with the desired value of more than 0.90, the root mean square error of approximation (RMSEA) with the desired value of less than 0.07, and the standardised root mean square (SRMR) with the desired value of less than 0.08. The same cut-off values of less than 0.08 for SRMR were given by [13,14]. Other fit indices that commonly presented are the Normed Fit Index (NFI) and the Non-Normed Fit Index (NNFI) with cut-off values of more than 0.90 and 0.95, respectively [15,16,17]. Items with factor loading less than 0.40 were inspected and were treated as problematic items. Problematic items were removed from the measurement model iteratively after adequate theoretical support was carried out by researchers.

Convergent validity was assessed using composite reliability (CR) and average variance extracted (AVE). CR calculated by Raykov’s method was applied to measure the reliability of the scale [18]. The recommended value of CR is 0.60 and above [19], and AVE is 0.50 and above [20]. Convergent validity is achieved when the item factor loadings are more than 0.50, CR and AVE values are above the recommended values [12]. Cronbach alpha was also reported in assessing the internal consistency of the factors of SCS-M. Cronbach alpha value of more than 0.60 was considered acceptable [21]. Discriminant validity was checked by examining the correlation between the factors in the final model. A correlation value of less than 0.85 was considered not too high and discriminant validity can be established [22]. Fornell and Larker suggested an alternate test of discriminant validity is by comparing the AVE for each factor with the squared correlations associated with that factor [20]. Discriminant validity is established if the AVE is more than the squared correlations [20].

## 3. Results

### 3.1. Demographic Characteristics

For demographic characteristics, there were five variables involved; gender, age, race, training frequency per week, and training period for each session. Table 1 showed the summary for the demographic characteristics.

### 3.2. Assumption Checking of CFA (Normality of Data Distribution)

Assumptions checking was performed prior to the CFA in LISREL. Table 2 shows the descriptive statistics of the 50 items and their values on skewness and kurtosis. The assumption of multivariate normality was not met based on Mardia multivariate skewness and kurtosis tests. The *p*-values for both tests were less than 0.05. According to Rosseel [23], “MLR—maximum likelihood parameter estimates with standard errors and a chi-square test statistic (when applicable) that robust to non-normality and non-independence of observations”. Hence, the MLR estimator was used for the subsequent CFA analyses.

### 3.3. Measurement Model of SCS-M

In the first measurement model (Model 1), 50 items of SCS-M were included in the CFA. We noticed that some of the fit indices were not within the acceptable range. Table 3 shows the model fit indices for the first measurement model with 50 items. Items with factor loading less than 0.50 were identified and inspected. Items which were found to be problematic were removed from the model iteratively. The model was re-analysed each time when an item was removed from the model. The final model consisted of 33 items.

Table 4 shows the standardised factor loading and Cronbach’s alpha for the final measurement model of SCS-M with 33 items. The Cronbach alpha values for all five factors were above 0.60, with acceptable reliability.

Table 5 shows the correlation values between the factors for the final measurement model of SCS-M, 33 items, which ranged between 0.39 to 0.70. The correlation values were below 0.85, which support the discriminant validity of the construct. In addition, all AVEs are greater than the corresponding interconstruct squared correlation estimates in Table 5. Thus, this test does not suggest problems with discriminant validity.

Table 5 shows the AVE and CR values for all the factors were above 0.50 and 0.70, respectively. The CR values exceed 0.70 suggesting adequate reliability for the SCS-M with 33 items. In addition, all factors loadings were above 0.50 and model fits relatively well. Thus, the evidence supports the convergent validity of the measurement model of SCS-M.

## 4. Discussion

The development of the SCS-M is crucial for assessing the courage levels in Malaysian Silat athletes. In the current study, a confirmatory analysis has been carried out to assess the factor structure of the SCS-M. Based on the previous study, the original version of the SCS was found to be reliable, valid, and stable over time [4]. The SCS is considered the latest and only available scale for measuring courage levels. Hence, in order to introduce the scale to local Malaysian athletes who speak and understand the Malay language, we translated the original English version of the SCS into a Malay version of the SCS-M.

There were several measurement models tested to obtain the most valid and reliable of the SCS-M. In the first measurement model tested, 50 items of the long version of SCS-M were included where some of the fit indices were not within the acceptable value. This is probably due to some of the items not being suitable for the current athletes’ condition or not applicable to Malaysians’ culture. The second measurement model was obtained after removal of problematic items (17 items) that can be considered not suitable for the Malaysian Silat athletes. The Cronbach’s alpha values were relatively not high, yet, the CR values showed that all factors produced a value of above 0.70, which was considered as an acceptable value of CR [24]. Our aim to determine the final best fit model was achieved on the final measurement model of SCS-M with 33 items, with acceptable fit indices values and composite reliability.

Some limitations were encountered during the execution of the current study. The most challenging limitation was the time constraint. Although the researchers had a few months to collect data, finding the right tournament and sporting events was very challenging. One condition that hindered the data collection during the study was the upcoming South-East Asian Games. Within those few months, the researchers could not meet the Silat athletes during their training periods, and researchers had to collect the data during the Silat tournament. Measuring the factors during the tournaments led the researchers to expect those athletes to exhibit optimum levels of courage. At different stages during the data collection, the researchers also dealt with time limitations. Distributing the questionnaires before the combat started was the ideal time, as athletes spent this time relaxing their bodies and calming their minds in their rooms and were able to take a few uninterrupted minutes to answer the questionnaires. However, due to unexpected circumstances, the researchers were able to distribute only a few questionnaires during this ‘ideal time’.

Another limitation faced by the researchers was the lack of co-operation from the managers, coaches, and athletes. Undeniably, some of them were co-operative, supportive, and enthusiastic upon receiving the questionnaires; however, others were not co-operative during this time and some teams ultimately refused to be involved in the study. Nonetheless, the researchers captured their stressful situations, such as limited preparation time, pressure dealing with anxiety and focusing on their combat techniques and strategies, and this time period proved to be the ideal time for measuring their courage. The researchers also presumed that the lack of co-operation might be triggered by thoughts that they would not benefit from participating in the study. As a consequence of time constraints, the researchers had limited opportunity to give each athlete a complete explanation about the importance of developing the relatively new field of sport psychology in our nation.

In the current study, validation of the Malay version of the SCS-M focused on studying Silat as the only sport. Conducting similar studies on other athletic disciplines is highly recommended. We suggest that future research focus on martial artists who reported possess higher psychological attributes compared to other disciplines. It is also suggested to do the studies for longer time periods so proper data collection methods can be planned to give better results than the current study.

## 5. Conclusions

The current study aimed to validate the translated Malay version of the SCS-M. To accomplish this objective, two types of statistical analyses were executed, CFA using LISREL 9.1 and Cronbach’s alpha using SPSS 24 software. At the end of the study, the findings showed that the results appeared to be acceptable for the measurement model of SCS-M with 33 items. The SCS-M with 33 items was considered valid and reliable to be used among Malaysian athletes, mainly Silat athletes, to determine their levels of courage.

## Figures and Tables

**Table 1:** Summary of the Silat athletes’ demographic characteristics.

Variables	*n* (%)	Mean (*SD*)
Age (years)		18.71 (2.631)
Gender		
Male	178 (66.7)	
Female	86 (33.3)	
Race		
Malay	246 (95.3)	
Chinese	-	
India	-	
Others (Ethnics in Sabah and Sarawak)	12 (4.7)	
Training frequency per week		5.20 (3.995)
Training period per session in minutes		139.92 (64.168)

*SD* = Standard deviation.

**Table 2:** Descriptive statistics, skewness, and kurtosis for the Malay language version of the Sports Courage Scale (SCS-M) 50 items.

Item	Mean	*SD*	Skewness	Kurtosis	Item	Mean	*SD*	Skewness	Kurtosis
S1	2.94	0.99	0.02	−0.24	S26	3.38	0.90	−0.41	−0.04
S2	3.57	0.90	−0.13	−0.29	S27	2.69	0.92	0.29	0.29
S3	3.14	0.79	0.17	0.69	S28	3.35	0.86	−0.03	0.13
S4	3.33	0.98	−0.32	−0.16	S29	3.56	0.90	−0.25	0.15
S5	3.74	0.86	−0.41	−0.02	S30	3.66	0.89	−0.47	0.28
S6	3.19	0.99	−0.36	−0.33	S31	3.70	0.92	−0.42	0.05
S7	3.74	0.863	−0.28	−0.20	S32	3.55	0.86	−0.21	−0.09
S8	3.52	0.93	−0.16	−0.18	S33	2.68	0.93	−0.02	−0.19
S9	3.65	0.91	−0.35	0.06	S34	3.53	0.99	−0.61	0.03
S10	3.15	0.80	−0.04	−0.21	S35	3.37	0.78	−0.06	0.03
S11	3.49	1.07	−0.26	−0.73	S36	3.67	0.89	−0.05	−0.35
S12	3.14	0.86	0.03	0.14	S37	3.74	0.88	−0.35	−0.02
S13	3.45	0.86	0.05	−0.28	S38	2.96	0.98	0.09	−0.24
S14	3.31	0.91	−0.20	−0.09	S39	3.66	0.89	−0.34	0.03
S15	3.41	0.84	−0.36	0.44	S40	3.53	0.83	−0.22	0.31
S16	2.78	0.90	0.16	−0.12	S41	3.23	0.90	0.16	0.06
S17	3.42	0.85	0.003	0.09	S42	3.34	0.97	−0.14	−0.29
S18	3.31	0.87	−0.14	0.29	S43	3.42	0.98	−0.14	−0.29
S19	3.67	0.85	−0.45	0.36	S44	3.25	0.95	−0.21	0.19
S20	3.52	0.87	−0.28	0.007	S45	3.48	0.95	−0.61	0.23
S21	2.67	0.99	0.14	−0.34	S46	3.00	0.94	0.03	−0.12
S22	3.59	0.94	−0.26	−0.33	S47	3.42	0.88	−0.03	0.04
S23	3.45	0.88	−0.20	0.05	S48	3.36	0.83	0.01	0.21
S24	2.78	0.77	−0.34	0.46	S49	3.36	0.85	0.00	−0.15
S25	3.48	0.85	−0.38	0.64	S50	3.23	0.95	−0.13	−0.14

*SD* = Standard deviation.

**Table 3:** Value of goodness-of-fit indices for first measurement model of SCS-M.

Model	RMSEA	CFI	NFI	NNFI	RMR	SRMR
Model 1 (50 items)	0.06	0.93	0.87	0.93	0.14	0.09
Model 2 (33 items)	0.06	0.94	0.89	0.94	0.05	0.07

**Table 4:** Standardised factor loading, Cronbach’s alpha of the final measurement model of SCS-M, 33 items.

Factors/Items	Standardised Factor Loading	Cronbach’s Alpha
Assertiveness		0.71
AT/S3	0.87	
AT/S8	0.73	
AT/S13	0.81	
AT/S18	0.79	
AT/S23	0.71	
AT/S26	0.64	
AT/S29	0.67	
Determination		0.76
DT/S2	0.76	
DT/S7	0.70	
DT/S17	0.80	
DT/S20	0.76	
DT/S22	0.60	
DT/S25	0.72	
DT/S28	0.76	
DT/S30	0.65	
Mastery		0.64
MT/S16	0.81	
MT/S21	0.68	
MT/S24	0.81	
MT/S27	0.69	
MT/S33	0.69	
Sacrifice behaviour		0.69
SB/S5	0.77	
SB/S10	0.84	
SB/S15	0.82	
SB/S31	0.62	
SB/S34	0.74	
SB/S39	0.75	
SB/S43	0.73	
Venturesome		0.68
VT/S4	0.85	
VT/S9	0.75	
VT/S14	0.83	
VT/S19	0.69	
VT/S32	0.66	
VT/S42	0.64	

The removal items (and their factors) are: S35 (Assertiveness); S12, S36 (Determination); S1, S6, S11, S38 (Mastery); S41, S45, S47, S49, S50 (Sacrifice behaviour); S37, S40, S44, S46, S48 (Venturesome).

**Table 5:** Composite reliability (CR), average variance extracted (AVE), and squared correlations.

Factors	CR	AVE	Factors				
			A	B	C	D	E
Assertiveness (**A**)	0.90	0.56	**1**	0.49	0.15	0.29	0.34
Determination (**B**)	0.90	0.52	0.70 *	**1**	0.19	0.37	0.27
Mastery (**C**)	0.86	0.55	0.39 *	0.44 *	**1**	0.20	0.20
Sacrifice behaviour (**D**)	0.90	0.57	0.54 *	0.61 *	0.45 *	**1**	0.44
Venturesome (**E**)	0.88	0.55	0.58 *	0.52 *	0.45 *	0.66	**1**

Values below diagonal are correlation estimates, * *p*-value < 0.005, values above diagonal are squared correlations.
